# Supplementary material for: Grain Boundary Engineering in 3D Porous Silver Electrocatalysts for Enhanced CO2-to-CO Conversion
Source: Molecules. 2025 Aug 24;30(17):3475. doi: 10.3390/molecules30173475 (PMC12430137; doi:10.3390/molecules30173475)
Supplement: Supplementary file 1 [file molecules-30-03475-s001.zip › molecules-3795760-supplementary.pdf]

Supporting Information

Grain Boundary Engineering in 3D Porous Silver Electrocatalysts for Enhanced CO_2-to-CO Conversion

Xiaoqian Xu [1], Song Yang [1], Yixiang Wang [1], Ying Chen [1], Assa Aravindh Sasikala Devi [2,3,*] and Feng Hu [1,*]

[1] Jiangsu Key Laboratory of Electrochemical Energy Storage Technologies, College of Materials Science and Technology, Nanjing University of Aeronautics and Astronautics, Nanjing 210016, China
[2] Research Unit of Sustainable Chemistry, Faculty of Technology, University of Oulu, 90014 Oulu, Finland
[3] Department of Physics, Durham University, Lower Mountjoy, South Rd, Durham DH1 3LE, UK
* Correspondence: assa.sasikaladevi@oulu.fi (A.A.S.D.); fenghu@nuaa.edu.cn (F.H.)

Contents

1. Figure S1. Flow chart of the Ag NPs and CP-Ag preparation.

2. Figure S2. (a) AgNP diameter distribution; (b) CP-Ag diameter distribution.

3. Figure S3. The SEM image of Ag NPs.

4. Figure S4. (a) CV plots of CP-Ag at different scan rates; (b) CV plots of AgNP at different scan rates.

5. Figure S5. The ^{1}H NMR spectra of the electrolyte after CO_2RR electrolysis for CP-Ag at −1.0 V vs. RHE.

6. Figure S6. The FE of CO and H_2 for (a) Ag foam; (b) AgNP.

7. Figure S7. The electrochemical impedance spectra of Ag foam, AgNP and CP-Ag.

8. Table S1. The key parameters of CO_2RR performance on CP-Ag and catalysts reported in previous literatures.

9. Table S2. The adsorption energies (E_{ads}) of Ag (100) and Ag (111).

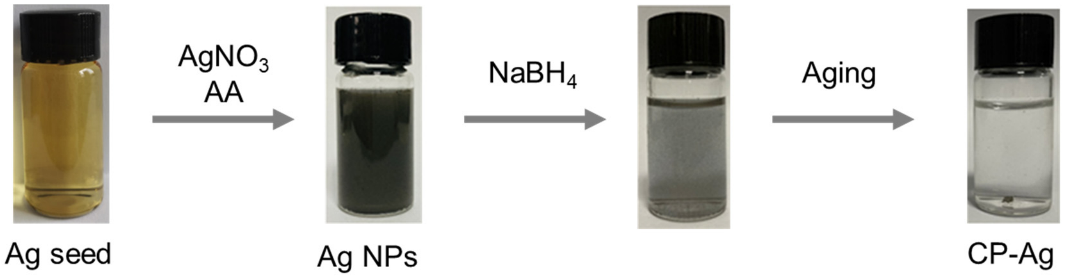

Figure S1. Scheme of the Ag NPs and CP-Ag preparation.

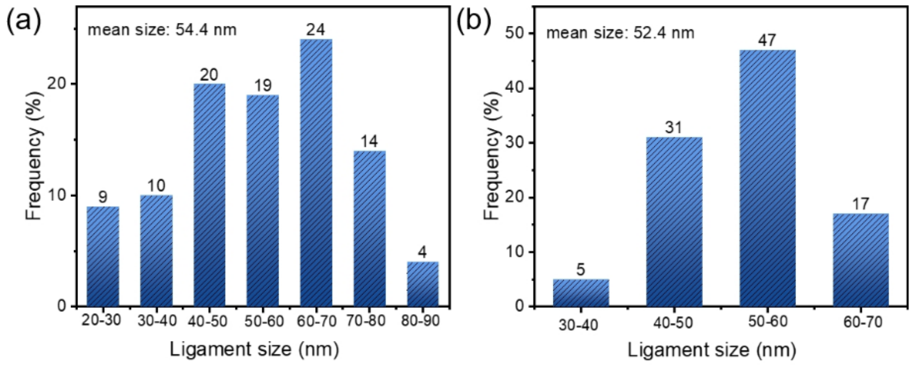

Figure S2. (a) AgNP diameter distribution; (b) CP-Ag diameter distribution.

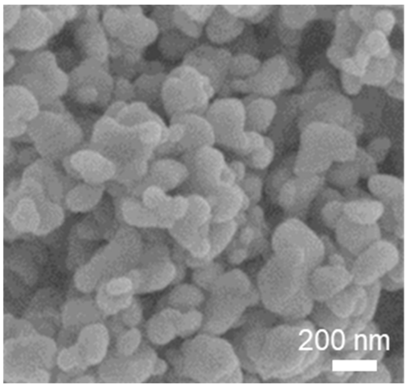

Figure S3. The SEM image of Ag NPs.

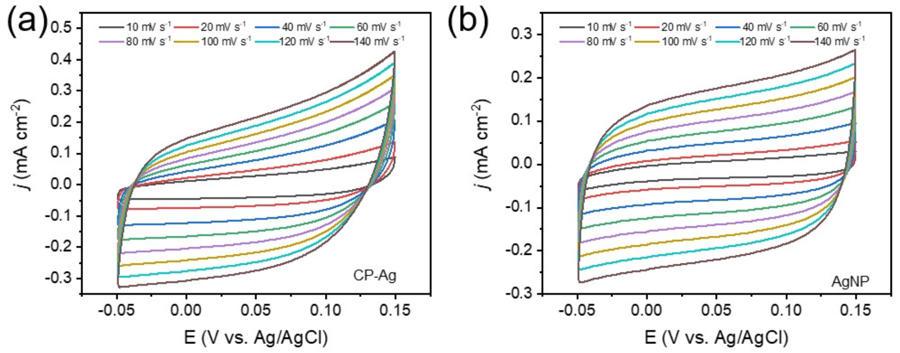

Figure S4. (a) CV plots of CP-Ag at different scan rates; (b) CV plots of AgNP at different scan rates.

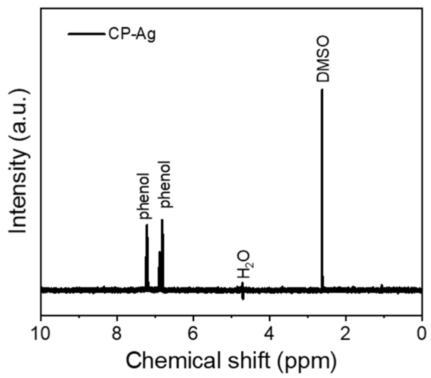

Figure S5. The ^1H NMR spectra of the electrolyte after CO_2RR electrolysis for CP-Ag at −1.0 V vs. RHE.

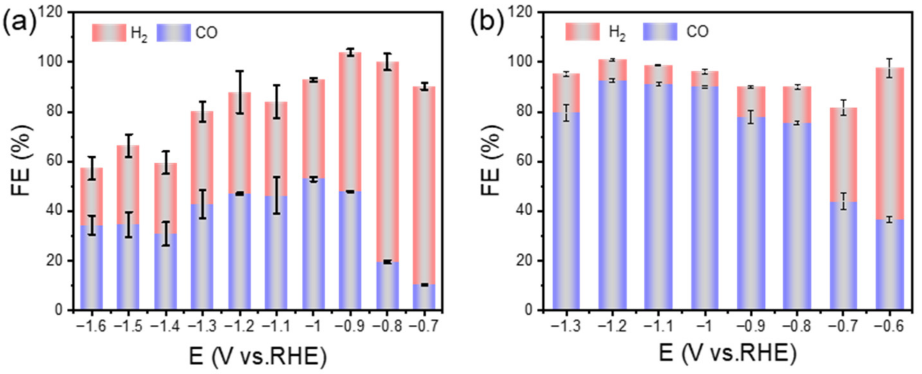

Figure S6. The FE of CO and H_2 for (a) Ag foam; (b) AgNP.

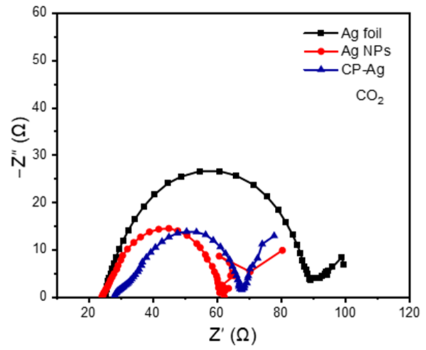

Figure S7. The electrochemical impedance spectra of Ag foam, AgNP and CP-Ag.

Table S1. The key parameters of CO_2RR performance on CP-Ag and catalysts reported in previous literatures.

catalysts	FE_{CO} (%)	j_{CO} (mA cm^{-2})	Potential (V vs. RHE)	electrolyte	stability	Ref.
CP-Ag	96.6	−4.6	−1.0	0.1 M $KHCO_3$	40h@−50 mA cm^{-2}	This work
CV-activated Ag electrode	96.6	−7.4	−0.7	0.5 M $KHCO_3$	8h@−1.0 V vs.RHE	Angew. Chem. Int. Ed. 2024, e202410932
Cu/Ag(S)	93	−2.9	−1.0	0.1 M $KHCO_3$	/	ACS Nano 2023, 17, 2387−2398
Ag/d-HOPG	~100	−1.2	−1.3	0.1 M $KHCO_3$	6h@−1.0 V vs.RHE	ACS Catal. 2023, 13, 15301−15309
Ni-Ag/PC-N	99.2	−12.6	−0.8	0.1 M $KHCO_3$	10h@−0.8 V vs.RHE	Chem. Eng. J. 2023, 476, 146556–146567
Ag_{32}	96.44	−5.8	−0.8	0.5 M $NaHCO_3$	15h@−0.8 V vs.RHE	Nano Res. 2022, 15(10): 8908–8913
Ag_1-G	79.2	−6.5	−0.7	0.5 M $KHCO_3$	10h@−0.95 V vs. RHE	Appl. Catal. B. 2022, 318, 121826–121836
Ag_1/MnO_2	95.7	/	−0.85	0.1 M $KHCO_3$	9h@−0.9 V vs.RHE	Angew. Chem. Int. Ed. 2021, 60, 6170–6176
Ag_{15}	95	−13	−0.6	0.5 M $KHCO_3$	10h@−0.75 V vs.RHE	Angew. Chem. Int. Ed. 2021, 60, 26136–26141
Cu_2Sb NA/CF	86.5	−6.5	−0.9	0.1 M $KHCO_3$	12h@−0.9 V vs.RHE	Nano Res. 2021, 14(8): 2831–2836
Ag NF	95	−33	−1.2	0.5 M $KHCO_3$	10h@−1.1 V vs.RHE	ACS Catal. 2020, 10, 1444−1453

Table S2. The adsorption energies (E_{ads}) of Ag (100) and Ag (111).

Configuration	Total energy (eV)	Adsorption energy (eV)
Ag (001)	−79.16	/
Ag (111)	−182.91	/
CO_2	−22.96	/
*COOH	−23.94	/
Ag (001) + CO_2	−101.88	0.23 eV
Ag (001) + *COOH	−104.67	−1.57 eV
Ag (111) + CO_2	−205.85	0.11 eV
Ag (111) + *COOH	−208.47	−1.62 eV
